# Adaptation of Methanogenic Inocula to Anaerobic Digestion of Maize Silage

**DOI:** 10.3389/fmicb.2017.01881

**Published:** 2017-09-28

**Authors:** Martyna Wojcieszak, Adam Pyzik, Krzysztof Poszytek, Pawel S. Krawczyk, Adam Sobczak, Leszek Lipinski, Otton Roubinek, Jacek Palige, Aleksandra Sklodowska, Lukasz Drewniak

**Affiliations:** ^1^Laboratory of Environmental Pollution Analysis, Faculty of Biology, University of Warsaw, Warsaw, Poland; ^2^Institute of Biochemistry and Biophysics, Polish Academy of Sciences, Warsaw, Poland; ^3^Institute of Genetics and Biotechnology, Faculty of Biology, University of Warsaw, Warsaw, Poland; ^4^Institute of Nuclear Chemistry and Technology, Warsaw, Poland

**Keywords:** inoculum source, anaerobic digestion, maize silage, biodiversity dynamics, methanogenic consortia

## Abstract

A well-balanced microbial consortium is crucial for efficient biogas production. In turn, one of a major factor that influence on the structure of anaerobic digestion (AD) consortium is a source of microorganisms which are used as an inoculum. This study evaluated the influence of inoculum sources (with various origin) on adaptation of a biogas community and the efficiency of the biomethanization of maize silage. As initial inocula for AD of maize silage the samples from: (i) an agricultural biogas plant (ABP) which utilizes maize silage as a main substrate, (ii) cattle slurry (CS), which contain elevated levels of lignocelluloses materials, and (iii) raw sewage sludge (RSS) with low content of plant origin materials were used. The adaptation of methanogenic consortia was monitored during a series of passages, and the functionality of the adapted consortia was verified through start-up operation of AD in two-stage reactors. During the first stages of the adaptation phase, methanogenic consortia occurred very slowly, and only after several passages did the microbial community adapts to allow production of biogas with high methane content. The ABP consortium revealed highest biogas production in the adaptation and in the start-up process. The biodiversity dynamics monitored during adaptation and start-up process showed that community profile changed in a similar direction in three studied consortia. Native communities were very distinct to each other, while at the end of the Phase II of the start-up process microbial diversity profile was similar in all consortia. All adopted bacterial communities were dominated by representatives of *Porphyromonadaceae*, *Rikenellaceae*, *Ruminococcaceae*, and *Synergistaceae*. A shift from low acetate-preferring acetoclastic *Methanosaetaceae* (ABP and RSS) and/or hydrogenotrophic *Archaea*, e.g., *Methanomicrobiaceae* (CS) prevailing in the inoculum samples to larger populations of high acetate-preferring acetoclastic *Methanosarcinaceae* was observed by the end of the experiment. As a result, three independent, functional communities that syntrophically produced methane from acetate (primarily) and H_2_/CO_2_, methanol and methylamines were adapted. This study provides new insights into the specific process by which different inocula sampled from typical methanogenic environments that are commonly used to initiate industrial installations gradually adapted to allow biogas production from maize silage.

## Introduction

Since the 1990s, anaerobic digestion (AD) has emerged as one of the most effective and sustainable methods to limit the harmful effects of organic waste on the environment, reducing its disposal in landfills. Simultaneous to the reduction of organic content, AD processes generate a substantial amount of methane-rich biogas, which constitutes a promising fuel for renewable energy production. Biogas generation in AD allows complete recycling of various waste materials, including wastewater, industrial food waste, or animal manure, as well as energy crops, which are a valuable source of organic matter for biogas production. For example, maize is considered to have the highest yield potential due to its high content of dry matter (Oslaj et al., 2010; Tyagi and Lo, 2013).

Anaerobic digestion is a multistep process carried out by a number of specialized microorganisms which catalyze (i) the liquefaction and hydrolysis of insoluble organic compounds, (ii) the gasification of intermediates, and (iii) the mineralization and humification of organic matter. All of the stages of AD process: hydrolysis, acidogenesis, acetogenesis, and methanogenesis are strictly interrelated and the proper balance between growth and activities of particular group of microorganisms is crucial for high efficiency (Ali Shah et al., 2014). For example, activity of hydrolytic bacteria determines the rate and performance of other group of microorganisms involved in AD. Low rate of hydrolysis of lignocellulose results in the slowdown of the entire process of plant biomass degradation, thus leading to the reduction of the efficiency of biogas production (Sun and Cheng, 2002). It is also known that for stable and efficient biogas production a strict cooperation between syntrophic bacteria and methanogenic archaeon’s is required. Excess of hydrogen produced by acetogenic bacteria can be toxic to them, therefore symbiosis with hydrogenotrophic archaea is required (Ali Shah et al., 2014). For this reason one of the key factors that directly influence on biogas yields is the selection and the use of the inoculum, which contain the appropriate groups of microorganism capable interacts with each other and able to adapt to various environmental conditions.

The most common practice in full-scale biogas plant systems, which allows selecting and using the most appropriate AD inoculum, is to obtain a starter microbial community from another, already running AD plant reactor. Alternatively, cow, poultry, or piggery dung is used as a source of methanogenic microorganisms (Dhamodharan et al., 2015). These biomass materials are rich in different groups of anaerobic microorganisms, and, during natural selection in new feedstock, the proper biogas-community is formed. However, stable and effective biogas production takes longer to achieve when starting up AD with such inoculum than when using inoculum from other well-performing biogas plants. Various batch experiments have shown that the use of inocula from different origins may vary the efficiency of the methanization process of the same specific substrate (e.g., corn stover, wastewater sludge, etc.) (Lopes et al., 2004; Xu et al., 2012). Furthermore, lab-scale inoculation experiments confirmed that the use of an adapted microbial consortium can accelerate start-up of the digestion process (Goncalves et al., 2011; Hidalgo and Martin-Marroquin, 2014). Goncalves et al. (2011) showed a five-fold faster start-up of an olive mill wastewater treatment reactor with an oleate-adapted consortium compared to a non-acclimated consortium. Our previous paper showed that adapted hydrolytic microbial consortia may improve the efficiency of maize silage degradation, which is demonstrated by increased glucose and volatile fatty acids (VFAs) production and increased biogas yield (Poszytek et al., 2017). The results of selection of hydrolytic consortia also showed that substrate input was the main driving force responsible for the changes in the community structure.

Along with the origin of the inoculum, an important parameter of the AD process is the reactor operation, which can determine the microbial structure in long-term process, allowing the adaptation of inoculum to changing conditions (De Vrieze et al., 2015; Wilkins et al., 2015; Han et al., 2016). Among the factors/parameters in the reactor environment that directly influence on the growth, performance, and the community structure are primarily: temperature variations (Ho et al., 2014), organic loading rate (OLR) (Kundu et al., 2014), increased VFAs and ammonia concentration (De Vrieze et al., 2015).

Despite studies conducted in recent years, our knowledge about the microbial structure and adaptation process of inoculum is still poor, and we are not yet able to draw concrete conclusions about how anaerobic microbiome behave against environmental and process disturbances, and which microorganisms are required for optimal performance of reactors. To achieve this goal, we should broaden our knowledge in this area by comparing the multiple studies monitoring methanogenic populations in biogas reactors enriched with different substrates, operating under different conditions, and, most importantly, considering the source of inoculum.

Based on the above considerations, the first objective of the present study is to evaluate the influence of inoculum sources from an agricultural biogas plant (ABP), cattle slurry (CS), and raw sewage sludge (RSS) on the adaptation of a biogas-producing microbial consortium and biogas production yields when maize silage is used as the sole substrate in a series of batch culture experiments. ABP community was selected as a reference inoculum, which has been adapted for anaerobic degradation of maize silage on an industrial scale bioreactor for several of months. CS represents community that use lignocelluloses materials as one of the main nutrient substrate. In turn, RSS community served as source of physiologically and phylogenetically diversified inoculum, for which plant materials are merely an admixture to the main pool of digested organic matter. The second goal of this work is to reveal the microbial community structure and the biogas production during start-up experiments in a quasi-continuous, two-phase process using previously adapted inocula. The microbial community structure in both experiments was analyzed by sequencing of the bacterial and archaeal 16S rRNA gene amplicons.

## Materials and Methods

### Inocula and Substrates

Inocula were taken from environments that are specialized in AD and methane production, including: (i) a fermenter tank of an ABP in Miedzyrzec Podlaski, (Poland), fed with maize silage and operated at mesophilic temperatures, (ii) RSS from the municipal sewage treatment plant “Czajka” in Warsaw (Poland), and (iii) CS from a farm in Mikanow (Poland). Methanogenic inocula (comprised of solid and liquids) were sampled in a hermetic canister or container, transported to the laboratory and stored for a maximum of 16 h at the following temperatures: (i) 37°C for ABP and RSS or (ii) in 23°C for CS sample prior to cultivation experiments. To analyze the microbial community structure, 50 mL of each sample was centrifuged (8000 × *g*, 4°C, 15 min) and the pellets were directly used for metagenomic DNA extraction.

In all performed experiments, the bioreactors were fed with maize silage provided by a farm located in Mikanow, Poland. A bulk amount of maize silage was transported from Mikanow to the laboratory at room temperature, portioned into plastic bags, and stored at 4°C. The physico-chemical characteristics of the methanogenic inocula and substrate are shown in **Table 1**.

**Table 1:** Physico-chemical characteristics of the inoculum and maize silage.

Parameters	Units	Maize silage	Agricultural biogas plant (ABP)	Cattle slurry (CS)	Raw sewage sludge (RSS)
pH	–	3.77	7.35	7.45	6.00
TS	% FM	37.00	4.00	2.21	4.00
VS	% TS	96.00	70.93	45.60	64.81
COD	g/L	38.90	42.5	18.40	74.33
VFAs	g/L	1.05	7.44	11.30	11.93

*COD, chemical oxygen demand; TS, total solids; VFAs, volatile fatty acids; VS, volatile solid; FM, fresh matter*.

### Laboratory Reactors Operation

**FIGURE 1:**
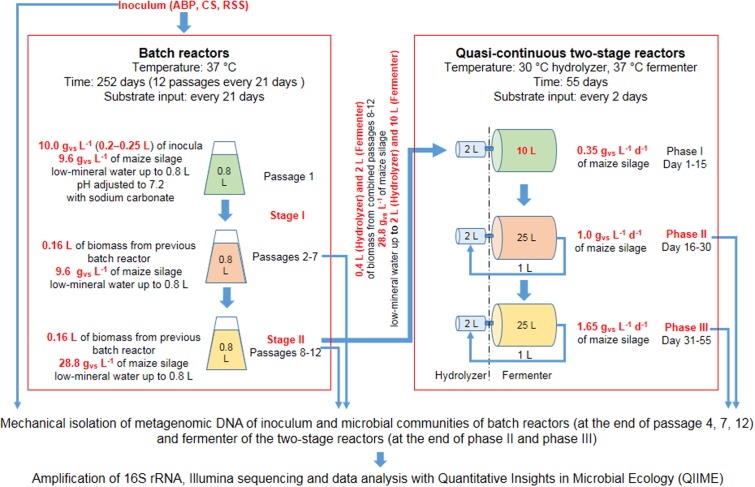
Scheme of the laboratory reactors operation.

Schematic visualization of laboratory scale experiments is shown in **Figure 1**. The preselection experiment was carried out in lab-scale bioreactors with a working volume of 800 mL, made of 1 L GL 45 glass bottles (Schott Duran, Germany) connected with Dreschel scrubbers and 1 L Tedlar gas bags (Sigma, Germany) as a biogas collector. The batch AD was conducted in triplicate. Batch cultivation was conducted until biogas productions in three successive passages were on the similar level and the methane content was above 60%. The similar biogas production with the high methane content was achieved in the second stage of adaptation (passages 8–12) (Supplementary Table S1).

The adapted consortia were then used in two-stage reactors to verify the procedure of scaling-up of the consortia volume and start-up enhancing properties for biogas production of the adopted microbial consortia. The remains of biomass from passages 8–12 were subsampled and further cultivated in batch reactors (in the same manner as for Stage II, **Figure 1**) in order to achieve sufficient amount of consortia required for inoculation of a two-stage biogas reactor used in the start-up experiment.

Two-stage bioreactor was constructed based on Polish Patent no. PL197595 (Krylowicz et al., 2001). The reactor was equipped with hydraulic agitation and operated in a quasi-continuous mode (**Table 2**).

**Table 2:** Operational conditions of the two-stage reactors during the three experimental phases.

Phase	Period (days)	HRT^a^ (days)	OLR^b^ (g_vs_/L/day)
I	1–15	12	0.35
II	16–30	28	1.00
III	31–55	28	1.65

*^a^Hydraulic retention time; ^b^Organic loading rate*.

Determination of the biodiversity of laboratory microbial consortia was performed on metagenomic DNA isolated from batch reactors (at the end of passage 4, 7, 12) and fermenter of the two-stage reactors (at the end of Phase II and Phase III).

### Analytical Methods

To control the AD process and characterize the initial inocula, the following parameters were determined: volume and composition of the biogas, VFAs, total solids (TS), volatile solids (VS), chemical oxygen demand (COD), total ammonia nitrogen (TAN), and pH. TS and VS analyses were performed according to standard methods described in the American Public Health Association [APHA] (1998) Standard Methods. VFAs, COD, and TAN were determined using Nanocolor^®^kits (Macherey-Nagel, Germany). The C and N elemental contents were quantified using a CHNS Elemental Analyzer EA1112 (Thermo Finnigan). Biogas production was monitored daily with a MilliGascounter MGC-1 (Ritter, Germany). Methane content was analyzed by Gas Chromatography Mass Spectrometry (GC-MS) (Agilent, United States) or with a gas analyzer GA5000 (Geotech, United Kingdom). The separation of biogas was performed using an Agilent 7890A Series Gas Chromatograph (GC) interfaced to an Agilent 5973c Network Mass Selective Detector (Agilent Technologies, United States). A gas sample was injected with split 1:500 (sample; carrier gas) by gastight injector to a HP-PLOT Q column (30 m × 0.32 mm I.D., 0.20 μm film thickness, Agilent Technologies, United States) using He as the carrier gas at 1 mL/min. The ion source was maintained at 250°C; the GC oven was programmed with a stable temperature 70°C (for 10 min). Mass spectrometry (MS) analysis was carried out in the electron-impact mode at an ionizing potential of 70 eV. Mass spectra were recorded from m/z 1 to 100 (0–10 min).

### DNA Extraction and 16S rRNA Gene Amplification

To analyze the microbial community structure at different stages of the experiment (inocula, adaptation, or start-up phase), 25–50 mL of each sample was centrifuged (8000 × *g*, 4°C, 15 min) and the pellet containing bacteria and plant debris was immediately transferred and stored on dry ice prior to DNA extraction. Metagenomic DNA was isolated according to the method described by Dziewit et al. (2015). Briefly, 1 g of centrifuged pellet (containing microbial cells) were disrupted with a 5-step bead-beating protocol, supplemented with freezing and thawing. Final DNA purification from protein, humic, and other substances was carried by CsCl density gradient ultracentrifugation. The concentration and quality of the purified metagenomic DNA was estimated using a NanoDrop 2000 instrument (NanoDrop Technologies) and gel electrophoresis.

The metagenomic DNA was used as a template for amplification of archaeal and bacterial hypervariable V3–V4 regions of the 16S rRNA gene with the following primers: S-D-Arch-0349-a-S-17/S-D-Arch-0786-a-A-20 (GYGCASCAGKCGMGAAW and GGACTACVSGGGTATCTAAT) and S-D-Bact-0341-b-S-17/S-D-Bact-0785-a-A-21 (CCTACGGGNGGCWGCAG and GACTACHVGGGTATCTAATCC), as described by Klindworth et al. (2013). The reaction mixture (50 μL) contained 100 ng template DNA and primers, and 0.02 U of Phusion High-Fidelity DNA Polymerase (Thermo Scientific).

Archaeal and bacterial 16S rRNA fragments were PCR-amplified in a TProfessional Thermocycler (Biometra) with 25 and 20 cycles, respectively. PCR conditions were as follows: initial denaturation (5 min at 96°C), cycles consisting of denaturation (30 s at 96°C), annealing (50 s at 54°C for *Archaea* and 58°C for *Bacteria*), extension (25 s at 72°C), and a final extension step (5 min at 72°C). The PCR products were analyzed by horizontal gel electrophoresis (2% agarose with ethidium bromide in 1x TAE) and then purified with Agencourt AMPure XP beads (Beckman Coulter).

### Sequencing Library Preparation and Amplicon Sequencing

To prepare libraries, approximately 250 ng of amplified DNA (pooled from the PCR replicates) was used with the Illumina TruSeq DNA Sample Preparation Kit according to the manufacturer’s protocol, except that the final library amplification step was omitted. Libraries were verified using the 2100 Bioanalyzer (Agilent) High-Sensitivity DNA Assay and KAPA Library Quantification Kits (Illumina).

Amplicon DNA sequencing was performed using the paired end Illumina MiSeq technology (MiSeq Illumina Kit V3) with a read length 2×300 bp. Computational analyses were performed in a similar manner as described in Nelson et al. (2012), using a local computing environment with the Quantitative Insights in Microbial Ecology (QIIME, v1.9.0) pipeline (Caporaso et al., 2010). Briefly, raw sequences were processed with the Cutadapt software enabling trimming of the nucleotides corresponding to the sequence of adapters and primers used for PCR amplification and library preparation. In a next step, sequences were merged and combined into a single fastq file, in order to ensure an even treatment and comparison QIIME analyses. This resulted in generation of 1.9 mln sequences with a mean length of 406 nucleotides (from 376 to 555 nt). Chimera detection was performed using usearch61 (Edgar et al., 2011) with subsequent filtering from sequences and *de novo* operational taxonomic unit (OTU) picking with uclust (Edgar, 2010) clustered at 97% similarity against the SILVA version 128 reference OTU alignment (Quast et al., 2013). A representative sequence for each OTU was selected and then the taxonomic assignment was made using the RDP Classifier v2.2 (Wang et al., 2007). Additional filtering for sequence errors was performed with the filter_otus_from_otu_table.py script by removing OTUs appearing in fewer than three samples and represented by less than 0.005% of the total sequences.

Taxonomic figures were prepared based on OTU tables specific for bacterial and archaeal amplicons, with a family level default. Sequences that were not assigned at the family level were named in accordance with the lowest taxonomy that can be assigned. A Principal Coordinates Analysis (PCoA) plot was constructed to visualize the dissimilarity of samples at different stages of the experiment.

Raw sequences obtained in this study were deposited in the SRA (NCBI) database under accession number PRJNA312575.

## Results

### Reactors Performance

#### Adaptation of the Microbial Consortia

The adaptation of specialized methanogenic microbial consortia from the three inocula that had a similar initial size (10 g_vs_/L) was carried out on a fresh substrate sample (9.6 g_vs_/L maize silage) until the methane content in each culture reached to ˜60% with similar level of biogas production (Supplementary Table S1). During the first three passages (9 weeks of cultivation), biogas production from maize silage was observed for all consortia, and the cumulative volume were 149.53 L/kg_vs_, 142.33 L/kg_vs_, and 121.7 L/kg_vs_, for ABP, CS, and RSS, respectively. As expected in these early stages, the best biogas quality (49% of CH_4_) was observed for ABP consortium (sampled from a stably running industrial biogas plant reactor fed with maize silage). Whereas, biogas from RSS and CS consortia contained only 15% and 19% of methane, respectively (data not shown).

During the passages 4–7 (week 10–21 of the experiment), the average methane content in the biogas increased. For the ABP community, improvement in biogas quality (only 4%) only reached 53%, but in the CS bioreactor the methane concentration nearly doubled, to 34%, and for RSS it even tripled, reaching 48%. The cumulative volume of biogas yield with ABP, RSS, and CS inocula reached 325.49 L/kg_vs_, 264.14 L/kg_vs_, and 281.20 L/kg_vs_, respectively. The gradual increase in biogas yield and quality seen for all three consortia highlighted the ongoing process of community reorganization and adaptation for maize degradation. At this step of the AD process, physico-chemical parameters such as VFAs, COD, and TAN concentration were determined at the end of each passage (after 21 days of cultivation). At the end of each batch AD with different inoculum, the physico-chemical parameters were on the similar level, with the VFAs concentration ranging from 2.13 to 2.60 g/L, and with COD values between 5.5 and 7.2 g/L. Meanwhile, the TAN concentrations remained low (18.96–22.90 mg/L) in all reactors (**Table 3**).

**Table 3:** Physico-chemical characteristics of the anaerobic digestion process.

Parameters	Units	ABP		CS		RSS	
		4–7	8–12	4–7	8–12	4–7	8–12
CH_4_ content	%	52.63 ± 11.23	58.98 ± 2.91	34.04 ± 10.71	65.40 ± 3.68	47.93 ± 19.81	68.00 ± 1.08
Biogas production	L/kg_vs_	325.49 ± 39.39	499.42 ± 9.66	281.20 ± 59.53	489.90 ± 10.90	264.14 ± 50.93	386.17 ± 23.82
pH	–	6.10 ± 0.50	6.88 ± 0.37	6.00 ± 0.56	6.95 ± 0.38	6.22 ± 0.58	7.19 ± 0.31
COD	g/L	5.50 ± 0.45	6.40 ± 0.83	7.07 ± 0.78	5.20 ± 1.05	7.20 ± 0.70	5.30 ± 0.80
VFAs	g/L	2.13 ± 0.35	3.10 ± 0.73	2.24 ± 0.18	2.58 ± 0.92	2.60 ± 0.44	2.48 ± 0.58
TAN	mg/L	22.90 ± 1.92	15.80 ± 3.73	21.00 ± 0.85	1.10 ± 0.20	18.96 ± 1.36	147.40 ± 17.45

*Average parameters of the results received from passage 4–7 or 8–12, measured on the 21st day after each passage. COD, chemical oxygen demand; TAN, total ammonia nitrogen; VFAs, volatile fatty acids*.

In the second stage of the adaptation process (passages 8–12, weeks 22–36), the microbial consortia were fed with increased amounts of maize silage (up to 28.8 g_vs_/L). Methane concentration in the produced biogas reached 59% (ABP), 65% (CS), and 68% (RSS) (**Table 3**), and the accumulated volume of biogas at the end of passage 12 was 499.42 L/kg_vs_, 489.9 L/kg_vs_, and 386.17 L/kg_vs_, respectively. The TAN concentration was measured to be at low levels in all reactors, below 200 mg/L. Similar to the first step of adaptation, the VFAs and COD concentrations remained low and stable. VFAs concentrations ranged between 2.48 and 3.10 g/L, and the COD value was between 5.2 and 6.4 g/L (**Table 3**).

Agricultural biogas plant seems to be the best consortium for AD of maize silage (compared to CS and RSS inoculum). Only ABP consortium was able to biogas production above 300 L/kg_vs_ with methane concentration above 50% in first stage of adaptation process. Moreover, in second stage of adaptation the ABP consortium revealed the higher biogas production than in reactors with inoculum CS and RSS.

#### Start-up Operation of Anaerobic Digestion in a Two-Stage Reactor

**FIGURE 2:**
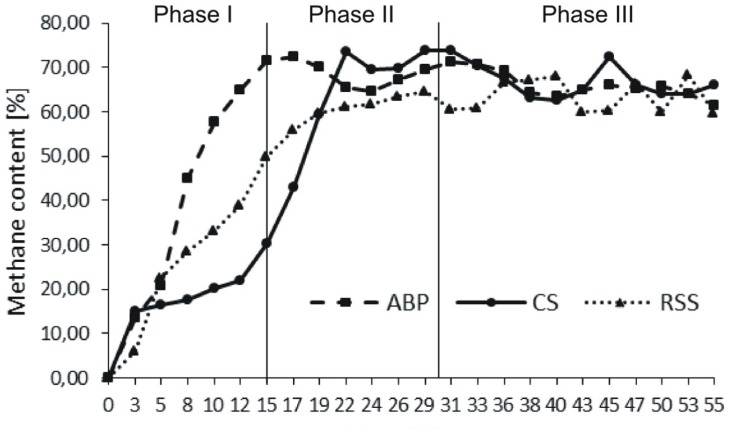
Methane content in biogas produced during anaerobic digestion in two-stage reactors.

The microbial communities adopted in one-stage, batch feed laboratory bioreactors were used in a subsequent phase of the experiment where we tested, if the adapted consortia would increase the rate of the start-up procedure of two-stage reactors where maize silage hydrolysis and methanization are separated. For this purpose, bioreactors were built with a hydrolyser of 2 L working volume separated from a fermenter of 25 L capacity. The reactors were inoculated with previously adapted methanogenic microbial consortia (ABP, CS, and RSS) coming from passages 8–12. The OLR increased gradually from Phase I to Phase III of start-up procedure 0.35–1.65 g_vs_/L/day, respectively (**Table 2**). During this experiment, physical and chemical parameters like biogas production and methane content were monitored (**Figure 2** and **Table 4**).

**Table 4:** Physico-chemical characteristics of anaerobic digestion.

Parameters	Units	ABP			CS			RSS		
		I	II	III	I	II	III	I	II	III
CH_4_ content	%	45.57 ± 23.86	68.21 ± 2.96	66.11 ± 3.08	20.29 ± 5.54	64.89 ± 11.99	66.88 ± 3.83	25.64 ± 14.95	61.11 ± 3.07	63.51 ± 3.75
Daily biogas production	L/kg_vs_	52.58 ± 17.24	37.92 ± 11.95	27.12 ± 1.87	21.11 ± 10.52	20.22 ± 3.65	21.69 ± 6.10	36.92 ± 27.91	17.85 ± 6.42	14.35 ± 1.97
pH	–	7.19 ± 0.52	7.47 ± 0.35	7.8 ± 0.07	7.13 ± 0.16	7.29 ± 0.05	7.88 ± 0.08	7.54 ± 0.15	7.85 ± 0.07	7.7 ± 0.16
COD	g/L	8.88 ± 2.16	3.70 ± 0.25	3.80 ± 0.36	8.75 ± 2.01	6.40 ± 0.44	6.90 ± 0.22	5.40 ± 2.02	7.04 ± 0.96	8.40 ± 0.70
VFAs	g/L	2.60 ± 0.25	2.07 ± 0.34	2.10 ± 0.83	4.48 ± 0.58	3.42 ± 0.38	3.52 ± 0.36	2.6 ± 0.30	1.64 ± 0.24	4.32 ± 0.11
TAN	mg/L	79.00 ± 15.17	100.30 ± 8.07	80.33 ± 7.51	112.00 ± 12.95	142.66 ± 6.43	113.00 ± 3.00	108.00 ± 25.14	41.66 ± 12.13	107.33 ± 3.06
C:N	–	21:01	15:01	10:01	33:01:00	20:01	14:01	39:01:00	20:01	15:01

*Average parameters of results achieved during each of the operation phases in the two-stage reactor. COD, chemical oxygen demand; TAN, total ammonia nitrogen; VFAs, volatile fatty acids*.

During Phase I of the start-up procedure (1–15 days), the maize silage concentration was at the same level as that in the batch experiment (passage 1–7), 9.6 g_vs_/L. Under these conditions, the biogas production in Phase I was unstable in each bioreactor. The average of daily biogas production in the first phase was 52.58 ± 17.24 L/kg_vs_ for ABP, 21.11 ± 10.52 L/kg_vs_ for CS, and 36.92 ± 27.91 L/kg_vs_ for RSS. The daily biogas production in Phase II reached to more stable level than in Phase I and the average biogas production was 37.92 ± 11.95 L/kg_vs_, 20.22 ± 3.65 L/kg_vs_, and 17.85 ± 6.42 L/kg_vs_, respectively. In the Phase III, further stabilization of the process was observed, as fluctuations between individual measurements points were ∼10%. The average daily biogas production during the last phase of operation was 27.12 ± 1.87 L/kg_vs_ for ABP, 21.69 ± 6.10 L/kg_vs_ for CS, and 14.35 ± 1.97 L/kg_vs_ for RSS (**Table 4**). The methane concentration analysis revealed that the ABP-adopted consortium needed only 15 days to start-up production of high methane content biogas (highest observed, 72%), while the CS and RSS bioreactors reached a similar level by day 20 (CH_4_ content 74% and 61%, respectively). In Phase II of the start-up procedure (days 16–30), the observed maximum of methane content was 72% for ABP, 74% for CS, and 64% for RSS (**Figure 2**). What was most important, the average methane content during the entire Phase II of the start-up phase was 68% (ABP), 65% (CS), and 61% (RSS), which is considered to be good CH_4_ levels desired by industrial biogas plants. During Phase III (days 31–55), the methane concentration was stable and exceeded 63% in all of the reactors (**Table 4**). The biogas quality evolution during each phase corresponds to a decline of daily biogas production during the start-up operation. The higher biogas production in Phase I was due to CO_2_ overproduction in the start-up phase (data not shown). At the end of start-up operation of two-stage reactors, all methanogenic consortia were able to stable biogas production with high methane concentration (especially consortium ABP).

In this study, physico-chemical parameters were also monitored. During start-up phases pH value were 7.13–7.88 in all reactors. The TAN concentrations in all reactors were below 200 mg/L. In reactor ABP, CS, and RSS, the VFAs concentration were between 2.10 and 2.60, 4.48 and 3.42, and 4.32 and 1.64 g/L, respectively. Only in reactor RSS, the VFAs and COD concentration were significantly increasing during start-up process. The lower biogas production in reactor RSS in Phase III (14.34 L/kg_vs_) (compared to reactor ABP – 27.12 L/kg_vs_) corresponding with higher concentration of VFAs and COD. The higher VFAs and COD concentrations in reactor RSS showed that the microorganisms consortia in reactor RSS could not effectively convert the organics into biogas.

#### Characterization of Microbial Communities

Microbial adaptation to methane fermentation form maize silage, was determined based on the analysis of 16S rRNA amplicons. The analysis of microbial dynamics of the selected methanogenic consortia was performed for three steps: (i) inoculum; (ii) adaptation to maize silage (passages 4, 7, 12); and (iii) start-up operation in a two-stage biogas reactor (Phase II and Phase III) (see Materials and Methods).

#### Bacterial Diversity

**FIGURE 3:**
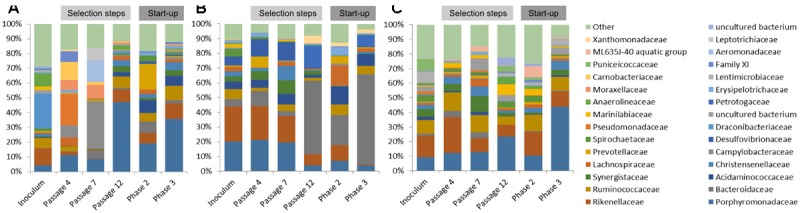
Relative abundance of bacterial operational taxonomic units (OTUs). Only those bacterial families with an abundance >5% in at least one sample are shown. **(A)** ABP, agricultural biogas plant, **(B)** RSS, raw sewage sludge, **(C)** CS, cattle slurry.

Native communities used for the laboratory cultivation was very distinct to each other. Most of the sequences of ABP consortium were assigned to *Draconibacteriaceae* (24%), followed by families *Rikenellaceae* (12%), *Anaerolineaceae* (9%), and *Ruminococcaceae* (7%). In the case of CS inoculum, *Pseudomonadaceae* (21%) was found to be the most predominant family, followed by families *Carnobacteriaceae* (12%), *Porphyromonadaceae* (11%), *Campylobacteriaceae* (8%), *Moraxellaceae* (7%), Family XI (7%), and *Lachnospiraceae* (6%). Finally, the RSS sample consisted mainly of *Campylobacteraceae* (32%), *Aeromonadaceae* (15%), *Leptotrichiaceae* (9%), *Porphyromonadaceae* (9%), *Moraxellaceae* (9%), and *Bacteroidaceae* (5%) bacteria families (**Figure 3**).

After cultivation in laboratory reactors, at the end of passage 4, we observed significant increase of *Porphyromonadaceae* family in all of the studied samples, which accounted for 47% (ABP), 19% (CS), and 36% (RSS) of total microbial structure. Moreover, all three samples were abundant in sequences assigned to *Rikenellaceae* (9%, 7%, and 10%) and *Ruminococcaceae* (8%, 6%, and 10%) for the ABP, CS, RSS, respectively (**Figure 3**). In the case of laboratory consortia originated from CS and RSS, bacteria family which exceeded 5% of total community structure was also *Acidaminococcaceae* 9% (CS) and 7% (RSS). Furthermore, CS community was highly enriched in *Prevotellaceae* (18%) and *Bacteroidaceae* (8%) compared to ABP and RSS samples where they accounted for less than 2% of total microbial community. By the end of passage 7, in all three studied consortia, the dominant family became *Porphyromonadaceae* (21% 20%, 20%) and *Rikenellaceae* (24%, 23%, 18%) followed by *Desulfovibrionaceae* (6%, 12%, 13%), *Acidaminococcaceae* (5%, 5%, 7%) and *Bacteroidaceae* (5%, 10%, 4%), ABP, CS, and RSS, respectively (**Figure 3**). However, there were also significant differences in the abundance of families such as *Christensenellaceae* (3%, 2%, 10%), *Prevotellaceae* (1%, 8%, 0%), *Spirochaetaceae* (5%, 2%, 0%), *Synergistaceae* (3%, 6%, 9%), and *Ruminococcaceae* (7%, 2%, 5%) ABP, CS, and RSS, respectively. At the end of the selection process in batch reactors, namely passage 12, the most predominant family was *Bacteroidaceae* which accounted for 50% (ABP), 20% (CS), and 61% (RSS). Families *Porphyromonadaceae* and *Rikenellaceae*, which were dominant in previous passages, diminished at least two-fold to the level of 4% and 8% in ABP sample, 7% and 11% in CS sample, 4% and 1% in RSS sample, respectively. Moreover, there were several bacterial families which had high abundance at passage 12 in certain samples while in others they accounted for less than 2%. These families were *Petrotogaceae* in ABP (15%) and RSS (8%), *Acidaminococcaceae* in CS (12%) and RSS (8%), *Ruminococcaceae* in CS (7%) and RSS (6%), *Lachnospiraceae Erysipelotrichaceae*, *Prevotellaceae* in CS (14%, 6%, 5%, respectively), and *Xanthomonadaceae* in ABP (6%).

**FIGURE 4:**
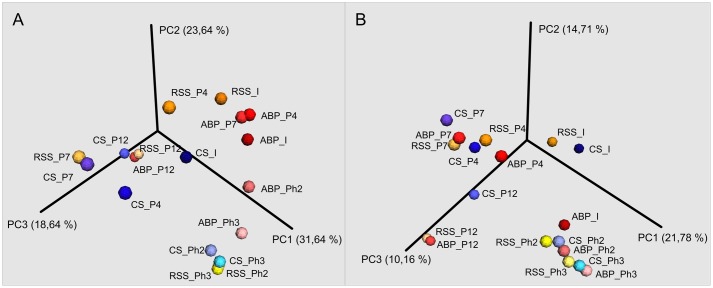
Principal Coordinates Analysis (PCoA) of Bray–Curtis dissimilarity of archaeal **(A)** and bacterial **(B)** diversity of studied samples: ABP, agricultural biogas plant. CS, cattle slurry; RSS, raw sewage sludge, representing microbial community at different stage of the experiment; I, Inoculum; P, Passage; Ph, Phase.

The microbial communities adopted in one-stage, batch feed laboratory bioreactors were used in a subsequent phase of the experiment where we tested if the adapted consortia would increase the rate of the start-up procedure of two-stage reactors where maize silage hydrolysis and methanization are separated. Biodiversity analysis at the end of Phase II of the start-up, showed that the microbial profile was similar in all three studied consortia (**Figure 4**) with predominance of *Porphyromonadaceae* (9%, 12%, 13%), *Rikenellaceae* (15%, 24%, 10%), *Ruminococcaceae* (9%, 13%, 12%), and *Synergistaceae* (5%, 5%, 11%) for ABP, CS, and RSS, respectively. Bacterial community of ABP sample had also high abundance of WCHB1-69 (8%) and *Puniceicoccaceae* (8%) while in the other two samples these two bacterial groups accounted for less than 1%. Furthermore, RSS consortium was enriched in bacteria from order W27 (8%), *Christensenellaceae* (7%), and *Lachnospiraceae* (5%). By the end of the experimental period (end of Phase III), CS community was very similar (1–2% difference) to that from Phase II, except for reduced abundance of *Rikenellaceae* in favor of ML635J-40 aquatic group bacteria (7%). In the case of ABP and RSS sample, at least three-fold increase of *Porphyromonadaceae* was observed, to the level of 24% and 44%, respectively. Additionally ABP consortium was enriched in bacteria from *Marinilabiaceae* (7%), *Anaerolineaceae* (5%), and order BS5 (5%).

#### Archaea Diversity

**FIGURE 5:**
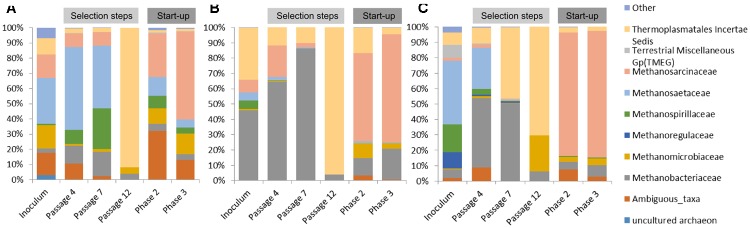
Relative abundance of archaeal OTUs. Only those families with abundance >1% in at least one sample are shown. **(A)** ABP, agricultural biogas plant, **(B)** RSS, raw sewage sludge, **(C)** CS, cattle slurry.

In the case of communities originating from ABP and RSS, the dominant archaeal group was *Methanosaetaceae* which accounted for 31% (ABP) and 42% (RSS), whereas CS was clearly dominated by *Methanobacteriaceae* (46%) and representative of *Thermoplasmatales Incertae Sedis* (34%). In both the ABP and RSS samples, there were also a significant number of sequences that could be classified as *Thermoplasmatales Incertae Sedis* (11% and 8%, respectively). It is also worth mentioning that the ABP sample had a large proportion of *Methanosarcinaceae* (16%), *Methanomicrobiaceae* (16%), and ambiguous taxa of *Bathyarchaeota* (15%), RSS sample had abundant *Methanospirillaceae* (18%), *Methanoregulaceae* (11%), Terrestrial Miscellaneous Gp (TMEG) (8%) and *Methanobacteriaceae* (5%), while CS sample *Methanosarcinaceae* (8%), *Methanospirillaceae* (6%), and *Methanosaetaceae* (5%) (**Figure 5**).

After the cultivation process, at the passage 4, most of the sequences were assigned to *Methanobacteriaceae* (12%, 65%, 45%), *Methanosaetaceae* (55%, 2%, 27%), *Methanosarcinaceae* (9%, 20%, 2%), *Thermoplasmatales Incertae Sedis* (3%, 12%, 10%), ambiguous taxa of *Bathyarchaeota* (11%, 0%, 9%), and *Methanospirillaceae* (9%, 0%, 4%), ABP, CS, RSS, respectively. Archaeal dynamics was analyzed next at the seventh passage, for which we observed further increase of the abundance of *Methanobacteriaceae* to the level of 16% (ABP), 86% (CS), and 50% (RSS). In the case of ABP, predominant was *Methanosaetaceae* (41%), *Methanospirillaceae* (27%), and *Methanosarcinaceae* (9%) while in the CS and RSS sample, we detected high representation of *Thermoplasmatales Incertae Sedis* (10% and 46%, respectively). At the end of the adaptation process (passage 12), a drastic shift in the *Archaea* community occurred to make the *Thermoplasmatales Incertae Sedis* the most predominant in all bioreactors (ABP 92%; CS 96%; and RSS 70%). For the RSS sample, the second most abundant archeon was *Methanomicrobiaceae* (24%).

Microorganisms selected in batch culture were then used in two-stage biogas reactors. Biodiversity analysis at the end of Phase II of the start-up, showed that predominant methanogen became *Methanosarcinaceae* which constituted 29% (ABP), 58% (CS), and 80% (RSS) of the archaeal community (**Figure 5**). Other *Archaea* which was abundant in at least one sample were also ambiguous taxa of *Bathyarchaeota* (32%, 2%, 7%), *Methanobacteriaceae* (5%, 11%, 5%), *Methanomicrobiaceae* (10%, 10%, 3%), *Thermoplasmatales Incertae Sedis* (2%, 16%, 4%), *Methanospirillaceae* (8%, 0%, 1%), *Methanosaetaceae* (12%, 1%, 0%), for ABP, CS, RSS, respectively. Following cultivation in laboratory reactors resulted in further increase of *Methanosarcinaceae* to the level of 58% (ABP), 70% (CS), and 82% (RSS) at the end of Phase III which was marked as the end of the experiment. At the same time, the richness of other archaeal families decreased, although abundance of some families increased for example *Methanobacteriaceae* to 20% (CS), 7% (RSS), and *Methanomicrobiaceae* to 13% (ABP).

## Discussion

This study aimed to evaluate the influence of inoculum sources on the adaptation of a biogas-producing microbial consortium and biogas production yields from maize silage in a series of batch culture experiments and to reveal the influence of previously adapted inocula on the microbial community structure during start-up experiments in a quasi-continuous, two-phase process. Biogas production and phylogenetic sequencing analysis revealed that the three different source of inoculum were able to gradually adapt to biogas production form maize silage. Moreover, microbial community analysis broadens knowledge about microbial community shift during the initial stages of digestion of maize silage.

The biogas production results reveled that during the first stages of the adaptation phase, methanogenic consortia occurred very slowly, and only after several passages did the microbial community adapts to allow production of biogas with high methane content (**Table 3**). The biogas yield and methane level in second steps of adaption reached values close to the maximum reported in the literature for one-step anaerobic degradation of maize silage. For example, methanization of maize silage described by Oslaj et al. (2010) produced a biogas yield ranging from 515 to 620 L/kg_vs_, and the methane content ranged from 55 to 58%. ABP consortium was firstly adapted to effective biogas production, because ABP inoculum was collected from industrial scale in which was used the same type of feedstock (maize silage).

During start-up AD, common physico-chemical parameters, biogas production and quality were monitored. The carbon to nitrogen (C/N) ratio is one of the important parameters influencing the digestion process. Many studies indicated that the optimal C/N ratios in methane fermentation were 20:1–30:1 (Puyuelo et al., 2011; Wang et al., 2014). TAN and VFAs also play a vital role in the performance and stability of AD. It is generally believed that TAN concentrations remain below 200 mg/L, thus they should not be considered as an inhibitor of the biogas production process (Rajagopal et al., 2013). VFAs can be accumulated during high organic loading, resulting in the decrease of pH and even the failure of AD (Wang et al., 2009; Zhang et al., 2014). The three reactors showed a similar increasing trend in methane production during the start-up phases (**Figure 2**). The reactor inoculated microbial consortium ABP already demonstrated after 15 days high quality biogas production (70% methane content), and the microbial communities CS and RSS adapted to produce biogas in the two-stage reactor after 20 days. During start-up phases of operation, two-stage reactors achieved the optimal parameters of C/N ratio in reactor ABP in Phase I and in reactors RSS and CS in Phase II (**Table 4**). The TAN concentration in all reactors was below 200 mg/L, therefore, it did not inhibit the process. In reactor ABP, VFAs concentration was stable between 2.07 and 2.6 g/L. In reactor RSS, VFAs accumulated to a concentration of 4.32 g/L, so they may have been the reason for the inhibition of biogas production in Phase III (**Table 4**).

Native communities used in this study differ from each other’s, both in the terms of *Bacteria* and *Archaea* biodiversity. This difference had direct impact of methane production at early stages of laboratory cultivation (passages 1–3) for which different values of methane concentration (ABP 49%, CS 19%, and RSS 15%) and biogas production (ABP 149.53 L/kg_vs_, CS 142.33 L/kg_vs_, and RSS 121.7 L/kg_vs_) were obtained. In the following passages 4–7, biogas yield doubled (ABP 325.49 L/kg_vs_, RSS 264.14 L/kg_vs_, and CS 281.20 L/kg_vs_) and methane concentration increased to 53% (ABP), 34% (CS), and 48% (RSS). At this point of the adaptation process, we observed increase of abundance of fermentative microorganisms such as *Porphyromonadaceae*, *Rikenellaceae*, *Bacteroidaceae, Ruminococcaceae*, and *Prevotellaceae*, which could indicate enhanced degradation of maize silage and substrate release as these microorganisms are described as VFAs and hydrogen producers (Kong et al., 2010; Mosoni et al., 2011; Ziganshin et al., 2011; Traversi et al., 2012; Stolze et al., 2015; Wegner and Liesack, 2016). Furthermore, at the passage 7, which marked the end of first stage of the adaptation process with 9.6 g_vs_/L of maize silage, we observed a significant increase of *Synergistaceae* and *Desulfovibrionaceae* for which the cumulative abundance reached 9% (ABP), 18% (CS), and 22% (RSS). These microorganisms are known to syntrophically interact with methanogens, for example by hydrogen transfer and thus can improve *Archaea* performance through hydrogenotrophic pathway (Vartoukian et al., 2007; Walker et al., 2012; Bretschger et al., 2015). In fact, our two studied samples of CS and RSS, *Archaea* community was dominated by hydrogenotrophic *Methanobacteriaceae* which indicates that adaptation process is toward methanogens that utilize H_2_ + CO_2_. In comparison, adaptation of ABP sample occurs more toward acetate utilization as the most predominant archaeal family was *Methanosaetaceae* which is adapted to low concentration of acetate (Liu and Whitman, 2008). What is noteworthy, despite significant differences in the initial biodiversity of analyzed samples, the bacterial population in each bioreactor seems to be changed in similar manners, whereas shifts among *Archaea* at this stage of the adaptation proceeded differently in all three samples (**Figure 4**).

After switching bioreactors to higher maize silage concentration (28.8 g_vs_/L), bacterial communities became dominated by *Bacteroidaceae* which suggests increased abilities to fermentative utilization of organic substrates (Chen et al., 2016). However, CS sample display higher versatility as the abundance of *Bacteroidaceae* was more even with other polysaccharide degraders, e.g., *Prevotellaceae, Lachnospiraceae*, *Rickenellaceae, Porphyromonadaceae*, and *Ruminococcaceae*. In turn, ABP and RSS sample were enriched in *Petrotogaceae*, which representatives are observed in anaerobic reactors where are involved in the fermentation of complex polysaccharides (Briones et al., 2007; Maus et al., 2016). What is more important is that, at passage 12 for all three studied communities, most of archaeal sequences (70–96%) were classified to group of *Thermoplasmatales Incertae Sedis.* This group was already abundant (46%) at passage 7 of RSS reactor. In all of the cases methane was still produced with good quality which indicates active methanogenesis, probably by utilization of methylated compounds (Borrel et al., 2012, 2014). However, further studies are needed to confirm this observation.

The microbial communities selected in adaptation process in one-stage, batch feed laboratory reactors were used for a start-up procedure of two-stage reactors. Bacterial structure of all three methanogenic consortia was again dominated by *Porphyromonadaceae*, *Rickenellaceae*, and *Ruminococcaceae* which shows their importance in the biogas system fed with maize silage. Moreover, analysis of *Archaea* biodiversity showed that *Methanosarcinaceae* outcompete other methanogens. This observation is in agreement with recent work of Goux et al. (2016). This indicates that *Methanosarcinaceae* it is better adapted to stable conditions of semi-continuous two-stage reactors as it is fast-growing and substrate-versatile methanogen which can utilize acetate, H_2_ + CO_2_ as well as methanol and methylamines for the methanogenesis (Liu and Whitman, 2008; De Vrieze et al., 2012). Interestingly, at Phase II, in the ABP sample approximately one third of archaeal sequences were classified to ambiguous taxa of *Bathyarchaeota* (formerly known as Miscellaneous Crenarchaeotic Group). Other studies suggests that these archeons may be involved in the degradation of complex organic matter and interaction with acetate-utilizing methanogens (Collins et al., 2005; Kubo et al., 2012). Our work also seems to confirm this suggestion as the ambiguous taxa of *Bathyarchaeota* and acetoclastic methanogens such as *Methanosaetaceae* and *Methanosarcinaceae* were abundant throughout cultivation of ABP community and the overall performance of biogas production was better than in other two studied consortia.

## Conclusion

The choice of the most suitable source of microorganisms to inoculate fermenters in biogas plants can have a tremendous influence on methane production and the efficacy of the entire installation. However, a common practice in the biogas industry is to inoculate fermenters with methanogenic samples without considering the given substrates, which in many cases can lead to insufficient methane production and can generate losses. This study provides new insights into the gradual adaptation of different inocula sampled from typical methanogenic environments that are commonly used to initiate industrial installations for biogas production from maize silage. The knowledge about adaption microbial community to biogas production from maize silage is important to understand microbial community shift during the initial stages of maize silage digestion. The adaptation process of methanogenic consortia during the first stages of the adaptation phases occurred very slowly, since only after several passages did the microbial community adapt to allow for the efficient production of biogas with high methane content. The start-up experiments showed that microbial communities that were previously adapted to a given substrate proved to be very effective inocula for new bioreactors, and could shorten the time until methane production began. The biogas production analysis revealed that ABP consortium was able to the highest biogas production in the adaptation and in the start-up process compared to consortia to CS and RSS. The high-throughput sequencing methods allowed us to follow changes in bacterial and archaeal biodiversity during the adaptation process. We observed a shift from acetoclastic methanogens (*Methanosaetaceae*, low-acetate preferring microorganisms) (ABP and RSS) and/or hydrogenotrophic *Archaea* (e.g., *Methanobacteriaceae*) (CS) that prevailed in the inoculum samples, to the dominance of high acetate-preferring acetoclastic methanogens (*Methanosarcinaceae*) at the end of experiment.

Based on available reference and our results, we concluded that archeons from *Thermoplasmatales Incertae Sedis* are likely methanogens which utilizes methylated compounds while the ambiguous *Bathyarchaeota* could be involved in methanogenesis process by syntrophic interactions with acetate utilizing methanogens. However, this observations need to be further investigated in experiment where concentration of specific intermediate substrates are highly controlled. Ideally microbial dynamics should be quantitatively measured by qRT-PCR experiments.

## Author Contributions

MW was involved in planning and executing adaptation and start-up experiments, DNA isolation, most of the chemical analyses, and in writing the manuscript. AP was involved in planning the metagenomics approach, isolating metagenomic DNA, deep sequencing, all bioinformatics analysis, and in writing the manuscript. KP participated in chemical analyses. PK participated in computational analysis. OR and JP constructed the two-stage bioreactors and participated in the start-up of the experiments. ASo and LL designed and supervised metagenomics and bioinformatics approaches, and helped draft the manuscript. ASk was involved in methodology and manuscript preparation, consultation, LD is the head of the project and directed microbial adaption, supervised biochemical analyses and was involved in consultation and article preparation. All authors read and approved the final manuscript.

## Conflict of Interest Statement

The authors declare that the research was conducted in the absence of any commercial or financial relationships that could be construed as a potential conflict of interest.
