# Supplementary material for: Adaptation of Methanogenic Inocula to Anaerobic Digestion of Maize Silage
Source: Front Microbiol. 2017 Sep 28;8:1881. doi: 10.3389/fmicb.2017.01881 (PMC5625012; doi:10.3389/fmicb.2017.01881)
Supplement: Supplementary file 1 [file Table_1.DOCX]

Supplementary Material

**Adaptation of methanogenic inocula to anaerobic digestion of maize silage**

Martyna Wojcieszak^1#^, Adam Pyzik^2#^, Krzysztof Poszytek^1^, Pawel S. Krawczyk^2^, Adam Sobczak^2,3^, Leszek Lipinski^2^, Otton Roubinek^4^, Jacek Palige^4^, Aleksandra Sklodowska^1^, Lukasz Drewniak^1*^

^1^ Laboratory of Environmental Pollution Analysis, Faculty of Biology, University of Warsaw, Ilji Miecznikowa 1, 02-096 Warsaw, Poland

^2^ Institute of Biochemistry and Biophysics, Polish Academy of Sciences, Adolfa Pawinskiego 5A, 02-106 Warsaw, Poland

^3^ Institute of Genetics and Biotechnology, Faculty of Biology, University of Warsaw, Ilji Miecznikowa 1, 02-096 Warsaw, Poland

^4^ Institute of Nuclear Chemistry and Technology, Dorodna 16, 03-195 Warsaw, Poland

# equal contributors, *corresponding author

**Corresponding author:**

Lukasz Drewniak

[ldrewniak@biol.uw.edu.pl](mailto:ldrewniak@biol.uw.edu.pl)

**Supplementary Table S1.** **Accumulated biogas production in second stage of adaptation**

| **Biogas production L/kg_vs_** | | | | | |
| --- | --- | --- | --- | --- | --- |
| **Passage** | **8** | **9** | **10** | **11** | **12** |
| ADP | 501.87 | 484.79 | 511.79 | 499.71 | 498.96 |
| CS | 487.74 | 492.89 | 496.96 | 472.15 | 499.74 |
| RSS | 352.02 | 389.17 | 397.16 | 410.14 | 387.34 |
